# Understanding the experiences of older adult participants and individuals involved in the delivery of a physical activity programme based on participatory approaches: A qualitative analysis

**DOI:** 10.1111/bjhp.12747

**Published:** 2024-09-23

**Authors:** Laura J. McGowan, Amy Davies, David P. French, Angela Devereux‐Fitzgerald, Elisabeth Boulton, Chris Todd, Christopher Phillipson, Rachael Powell

**Affiliations:** ^1^ Division of Psychology and Mental Health, Manchester Centre for Health Psychology, School of Health Sciences University of Manchester Manchester UK; ^2^ Manchester Academic Health Science Centre Manchester UK; ^3^ Division of Nursing, Midwifery and Social Work, School of Health Sciences University of Manchester Manchester UK; ^4^ Age UK London UK; ^5^ Applied Research Collaboration‐Greater Manchester, National Institute for Health and Care Research Manchester UK; ^6^ Manchester Institute for Collaborative Research on Ageing Manchester UK; ^7^ Manchester University NHS Foundation Trust Manchester UK; ^8^ School of Social Sciences University of Manchester Manchester UK; ^9^ Present address: NIHR Policy Research Unit in Behavioural and Social Sciences, Population Health Sciences Institute Newcastle University Newcastle Upon Tyne UK

**Keywords:** co‐design, co‐production, healthy ageing, intervention, physical activity, place‐based

## Abstract

**Background:**

The present study aimed to understand the experiences of older adult participants and service deliverers involved in a UK‐based physical activity programme, developed using participatory approaches.

**Methods:**

Focus groups and one‐to‐one interviews were conducted with 34 older adults (aged 55+ years) and 13 service providers. Inductive thematic analysis was conducted, structured using the framework approach.

**Findings:**

Four themes were identified: (1) Co‐designed activities met needs and encouraged attendance; (2) engagement and access of programme activities; (3) enjoyment and perceived benefits of sessions; and (4) support needs of individuals delivering activities. Co‐designed activities appeared to meet participant needs and instil a sense of ownership of the programme. Feeling able to relate to other participants seemed important and of potential relevance to attracting older adults to the programme. Peer support may help to increase confidence in attending sessions; place‐based approaches (using resources in local communities) and a flexible approach to involvement also seemed to facilitate engagement. Enjoyment of the programme appeared to be enhanced through activity variety and opportunity for socializing, with a sense of community being created through the support and encouragement of fellow participants. It was considered important that volunteers had appropriate recognition and ongoing support.

**Conclusions:**

These findings suggest that using participatory approaches may facilitate enjoyment and sustained engagement of older adults. Provision based on local community assets may contribute to sustainability of services. However, providing ongoing support is imperative, requiring further costs and resources over the longer‐term.

## INTRODUCTION

For older adults, physical activity improves well‐being, reduces chronic illness risk, and increases life expectancy (McPhee et al., 2016). Current United Kingdom (UK) recommendations state that adults aged 65 years and above should accumulate 150 min of moderate‐intensity physical activity each week, in addition to performing activities to improve muscle strength and balance on two and three days per week respectively (Department of Health and Social Care, 2019). This is in line with global recommendations (US Department of Health and Human Services, 2018; World Health Organization, 2010). However, most older adults fail to achieve this recommended amount of physical activity and physical activity decreases with age (Scholes & Mindell, 2013).

Interventions to promote physical activity in older adult populations have generally had smaller effects than interventions with younger adults, and these do not typically maintain increases in physical activity beyond 12 months (Hobbs et al., 2013; Murray et al., 2017; Stathi et al., 2022). These smaller effects in older adults may be because interventions are less acceptable and enjoyable to this population (French, Banafa, et al., 2020; French, Davies, et al., 2020). A systematic review of qualitative studies investigating older adults' views and experiences of participating in physical activity interventions found that many take part in such programmes for social and enjoyment‐related benefits, rather than a desire to increase physical fitness (Devereux‐Fitzgerald et al., 2016). A complementary systematic review exploring the views of older adults not involved in physical activity interventions found that older adults often considered physical activity as a by‐product of other more meaningful activities, rather than as a purposeful activity within itself (McGowan et al., 2018). This literature suggests that physical activity interventions may not align with older adults' own perceived needs and may therefore be of limited relevance to individuals.

Using participatory approaches in the development of physical activity interventions for older adults has been suggested as a way to increase acceptability in older adult groups and lead to sustained increases in physical activity (Haggis et al., 2013). Some recent evidence supports this contention (Stathi et al., 2022).

Participatory approaches typically involve a more collaborative approach to intervention design, development and implementation, whereby people designing interventions engage closely with stakeholders and members of the target population in all stages of the design process (Green, 2001; Israel et al., 1998). These collaborative approaches lend themselves to intervention development within community‐based settings as they empower the communities targeted by the intervention, and can promote understanding of culturally relevant issues (Wallerstein & Duran, 2006; Wells & Jones, 2006; Wieland et al., 2012). Types of participatory methods include co‐production, co‐design, place‐based working, and asset‐based perspectives. Varying descriptions have been used to define these approaches in the literature (Greenhalgh et al., 2016; Masterson et al., 2022), but commonly‐used definitions appear in Table 1.

**TABLE 1:** Definitions of participatory approaches.

Term	Definition
Co‐production	Participatory approaches that involve end‐users in intervention development, recognizing the value of their experiential knowledge as core to the success of development (Oliver et al., 2019)
Co‐design	The involvement of end‐users in identifying key problems to address and the best means of addressing them (as opposed to direct involvement in intervention development and delivery; Palmer et al., 2019)
Place‐based working	Considers the needs and assets of local communities, drawing on end‐users' experiential knowledge of their neighbourhoods and environments (Public Health England, 2019)
Asset‐based perspectives	Views end‐users as having important competencies as well as needs, and can therefore offer unique insight and may engage in non‐traditional, foreground roles within intervention organization and delivery, e.g. as champions or volunteers (Russell, 2011)
Strength‐based conversations	Focus on what individuals and communities can do for themselves, with the right support from the right people working alongside them (Social Care Institute for Excellence, 2015)
Community champions	People in the community who take on an issue or project and are committed to raising awareness and support for it (Government of Nova Scotia, 2012)

In developing physical activity interventions for older adult populations, participatory approaches share a common aim of engaging older adults in the creation of programmes and activities to increase physical activity. An overarching objective of such approaches is to develop activities based on the expressed needs of older adults in their local communities to ensure that programmes are valued in the locations where they are implemented. This may be particularly useful in lower socio‐economic areas, where there is a perceived lack of resources and unequal provision, and where older adults may feel unvalued (Devereux‐Fitzgerald et al., 2021). Participatory approaches may therefore increase the likelihood of physical activity programmes becoming a routine aspect of neighbourhoods, thus promoting longer‐term acceptability and sustainability (Collins et al., 2018).

However, there is a lack of evidence regarding the success of participatory approaches for increasing physical activity in older adults (Boulton et al., 2020). Moreover, most evaluations of co‐production approaches involve researchers as a key group in their production, alongside service providers and users (Kislov et al., 2018). It is therefore unclear how successful such participatory approaches will be without substantial researcher support, which is a major gap in evidence if such approaches are to become more routinely implemented outside of research settings. It is therefore timely to consider experiences with interventions created using participatory approaches.

The present research relates to the Greater Manchester Active Ageing (GM‐AA) programme, an initiative involving eight of the 10 Metropolitan Borough Councils (MBCs) that make up Greater Manchester, along with partner organizations being funded to develop innovative provision using participatory approaches. The initiative was designed to promote engagement of adults aged 55 years and above in physical activity across these eight MBCs within Greater Manchester (UK). Despite most national and international physical activity guidelines categorizing 65 years and above as older adulthood, low levels of physical activity are consistently prevalent in people aged 55 years and above (European Commission, 2018), and there is evidence of poor physical and mental health, as well as poor access to services to keep them well and active, in this age cohort (Boulton, 2024a, 2024b, 2024c). Furthermore, evidence shows that low levels of physical activity pre‐retirement are likely to be maintained post‐retirement (ter Hoeve et al., 2020), so it is important that recreational physical activity provision is available during pre‐retirement years. Notably, researchers were primarily involved in the evaluation of the programme, rather than contributing to its development as active partners.

Qualitative methods as part of a process evaluation can optimize learning from new services, as advised by the Medical Research Council (MRC) Framework for Developing and Evaluation Complex Interventions (Moore et al., 2015; Skivington et al., 2021). We have recently published a qualitative study evaluating experiences of participatory approaches in the design and development of the GM‐AA programme (Davies et al., 2021). Incorporating participatory approaches was viewed positively and perceived as highly valuable, although participants indicated that such approaches required significant time investment to do well. There was concern over sustainability due to short‐term funding cycles, but also the perception that asset‐based approaches led to older adults taking ownership of projects, which might then facilitate their continuation once funding stops. Importantly, Davies et al. (2021) showed that novel participatory approaches could be implemented in the design and development of physical activity programmes without substantial support from researchers.

The Davies et al. (2021) evaluation of the GM‐AA programme focused on how stakeholders experienced engaging with participatory approaches in the *design* and *development* of the programme. Indeed, most evaluations of using participatory approaches to date have focused specifically on the experience of employing participatory approaches in intervention development. By contrast, the present research evaluates *outcomes* of the participatory design, with particular focus on how those involved experienced the programme in terms of its enjoyability and acceptability (i.e. the ‘real‐world’ experience of the programme itself). Specifically, we aimed to explore the experiences of older adults who attended the GM‐AA programme activities, as well as individuals involved with programme delivery, and to understand the extent to which a programme developed using participatory methods could produce an acceptable intervention. Use of a multi‐perspective qualitative approach exploring the perspectives of both groups (older adult participants and service deliverers) in a single study enables related issues to be considered from different perspectives, providing a richer and more holistic insight into the overall phenomenon (Kendall et al., 2009). This multi‐perspective approach allowed us to not only investigate whether older adults found the programme activities acceptable but also to identify issues arising for those involved in delivery of services, and to obtain deliverers' perspectives on older adults who did not take up the intervention or participate in interviews.

## METHODS

Ethical approval was granted by the University Research Ethics Committee (UREC) at the University of Manchester, REC reference number 2018‐5001‐6798.

### Design and setting

A multi‐perspective qualitative cross‐sectional design was used; focus groups and semi‐structured interviews were conducted with GM‐AA service users and deliverers.

Greater Manchester is a large metropolitan area in England with a population of approximately 2.8 million people, and consists of a mix of high‐density urban areas, suburbs, semi‐rural and rural locations (Greater Manchester Combined Authority, 2017). It includes many highly deprived areas: six of the 10 MBCs rank within the 50 most deprived local authorities in England out of a total of 317, and a further two ranked within the most deprived 100 (Ministry of Housing, Communities, & Local Government, 2019). The older adult population in Greater Manchester is among the most ethnically diverse in England (Greater Manchester Combined Authority, 2024). Of residents aged 55 and older, 87% identify as White British. The second largest group, Asian British Pakistani, comprises 2.9%, followed by 1.5% identifying as Indian (Lang et al., 2024).

The GM‐AA programme was funded for £1 M ($1.4 M) over a two‐year period by Sport England with an explicit emphasis on the feasibility and impact of using participatory approaches to engaging older adults in physical activity. Selection of local authorities for funding was based on the extent to which their plans included consideration of their local needs and capacities and use of innovative participatory approaches. Activities were designed by stakeholders within each MBC; stakeholders were typically people from public health backgrounds or ageing professionals in local authorities working in partnership with people from leisure services, third sector organizations such as Age UK, and older adults living within the area where provision was to be targeted.

Across the eight MBCs there was a diverse range of 80 different activities (e.g. dancing, cycling, chair‐based exercise, walking netball, kayaking, tai chi, indoor curling) involving a mix of participatory approaches. An overview of approaches and activities developed across the eight localities is provided in File S1.

### Participants

Participants were older adults who were participating in the GM‐AA programme, and individuals involved in service delivery across the GM‐AA projects. The total number of participants included in the study was based on judgements of data adequacy (Vasileiou et al., 2018); this was based on representation of different MBCs, range of activity types, and the quality and sufficiency of data in relation to the research question and objectives.

Four focus groups were conducted with older adults attending activities running in four different MBCs across Greater Manchester. Groups were purposively selected to represent a range of activities across different MBCs. Selection across sites was important as different MBCs took different approaches to developing interventions and had different teams supporting intervention delivery. Participants in two focus groups took part in walking sports (Site 2, Site 4); the two other focus group members attended chair‐based exercise sessions (Site 1, Site 3). Focus groups were chosen as the data collection method for older adults attending activities in these sites so as to maximize the numbers of participant voices and ensure that a broad range of perspectives were obtained within each site with the time and resource available.

One‐to‐one interviews were conducted with older adults attending sessions at Site 5: a community location with indoor and outdoor facilities, providing activities such as yoga, table tennis, bowls and canoeing.

Interviews were conducted with people who were involved with delivering GM‐AA projects. In each MBC, 1–2 individuals who were involved in delivery were interviewed, and one further individual with a GM‐wide role was interviewed. We included people involved in delivery with a range of roles: active ageing co‐ordinators, physical activity instructors and volunteers. Co‐ordinators were involved in the delivery and operational side of project implementation (roles could include supporting instructors and older adult participants, facilitating data collection, managing budgets, carrying out consultation/co‐design work). Instructors were individuals commissioned to provide physical activity sessions to older adults. Volunteers were individuals who had an unpaid role in providing or supporting physical activity and could themselves be older adults.

### Data collection

Written informed consent was obtained from each participant prior to data collection. Topic guides containing open‐ended questions were used to guide focus groups and interviews. Topic guides were developed based on previous work within the topic area by the researchers (e.g. Devereux‐Fitzgerald et al., 2021) and adapted to the specific aims of the current study. Interviews and focus groups with older adults attending activities included questions concerning: what attracted them to the physical activity programme, what they thought about it, and about any perceived benefits from the group. For interviews with those delivering activities, topics included: experiences of working with the physical activity programme, aspects that might affect the success of the project, thoughts about what they might have done differently/recommendations for others, and feedback received from older adult participants. Example questions are provided in File S2. Interviews and focus groups took place between February 2019 and March 2020 and were conducted or facilitated by five members of the research team. Data collection took place in either a private room on the University campus, a private room within community buildings, or participants' own homes. All focus groups and interviews were audio‐recorded and transcribed verbatim. There were no monetary incentives for participation, however participants were reimbursed travel expenses.

### Analysis

An inductive approach was taken to focus on topics considered important to participants. Data from the different participant groups (older adults participating in the intervention and service deliverers) were analysed concurrently, in line with a multi‐perspective approach (Kendall et al., 2009), with equal weight given to all participants.

Thematic analysis was conducted within the data management structure of the Framework approach (Ritchie & Spencer, 1994; Spencer et al., 2014). The following steps were taken: (1) Familiarization—listening to recordings and repeated reading of transcripts. (2) Coding transcripts—initial codes were inductively generated from both groups' transcripts concurrently and collated into a single working framework, consisting of potential themes and sub‐themes. Due to similarities identified with our prior analysis in a related sample and publication (Davies et al., 2021), elements of this previous framework were used as a basis for the current analysis. (3) Indexing—the working thematic framework was applied to each transcript using a numerical structure for each category/code. (4) Charting—data were summarized into matrices, with each row representing a single participant, and columns representing the codes for each category. (5) Interpretation—authors reviewed the matrices to identify connections and patterns within and between participants and codes. Analysis of such patterns and interconnections resulted in the final set of themes and sub‐themes which are reported here.

The interviews with older adult participants were conducted earlier than focus groups for practical reasons. An initial analysis was conducted with these data, and findings compared with and incorporated into the analysis of the full data set.

## FINDINGS

Thirty‐four older adults participating in the GM‐AA programme took part in the study: 22 across four focus groups (Sites 1–4), and 12 in interviews (Site 5). Participants had a median age of 67 years (range from 50s to 80s); 25 (74%) were female, 9 (26%) male; all identified as white. Participant characteristics are largely reflective of those who enrolled in the GM‐AA programme, with 71% of those who provided demographic information (*n* = 1086) being female, and 87% describing their ethnicity as white (French, Davies, et al., 2020). Focus groups lasted between 49 and 84 min (mean = 67 min). Interviews lasted between 23 and 72 min (mean = 42 min).

Thirteen service deliverers were interviewed: three volunteers, four instructors, and six active ageing co‐ordinators. Their median age was 40 years (range from 20s to 70s); 10 were female, 3 male; most (12) identified as white, with one participant from an ethnic minority background. Interviews lasted between 29 and 86 min (mean = 47 min).

Four themes were identified: (1) Co‐designed activities met needs and encouraged attendance; (2) engagement and access of programme activities; (3) enjoyment and perceived benefits of sessions; and (4) support needs of individuals delivering activities. Themes 1–3 represent multi‐perspective themes, whereas theme 4 relates to the views of service deliverers only. An overview of themes and subthemes is shown in Table 2.

**TABLE 2:** Thematic overview of themes and subthemes.

Thematic overview			
Multi‐perspective themes (older adult participants and service deliverers)			Uni‐perspective theme (service deliverers only)
Co‐designed activities met needs and encouraged attendance	Engagement and access of programme activities	Enjoyment and perceived benefits of sessions	Support needs of individuals delivering activities
Co‐designed activities facilitated engagement Sense of ownership/responsibility towards group—self‐sufficiency Activities appropriate to ability Flexibility within sessions Awareness of instructors for need to work within individual's own abilities Negative response when felt level of activity beyond capabilities	Word of mouth through trusted/relatable source Older adults as assets—positive approach to engaging others Taking ownership/responsibility of marketing Competing commitments to engagement—pay as you go model helpful Having someone to attend with Peer mentor volunteers positively perceived Place‐based approaches—locality encouraged attendance Activities more targeted towards ‘older’ older adults—need to cater for ‘younger’ older adults	Variability and flexibility facilitated enjoyment Value of social aspects Sense of community support and encouragement of others Enjoyment enabled more activity Enjoyment of music in sessions	Value of high‐quality training for volunteers Increase peer contact of volunteers so do not feel alone—need support of others Empathy and listening skills of providers important Maintaining healthy boundaries Feeling under‐valued, need for recognition Personal circumstances impact ability to carry out roles

### Co‐designed activities met needs and encouraged attendance

Ensuring that the activities would fit the needs of individuals seemed key to determining engagement. Where consultation and/or co‐design with older adults took place in activity development, this appeared to be successful in ensuring activities met needs and encouraged attendance:

All the ideas that have come up have been the group's own ideas. I didn't want to put something out there for them and people over fifty‐five wouldn't enjoy it (P29, Co‐ordinator)

Like a co‐design approach with the participants and also the local residents, to find out what they want and I think that's encouraged local people to come along and take part. And we've seen a number of people from day one attend weekly throughout the last two years (P27, Co‐ordinator)

Involvement of service users in the initial design and development of the programme seemed to instil a sense of participant ownership and responsibility towards the group, resulting in a positively perceived level of self‐sufficiency:

I think moving forward it will be the likes of the organisers within the group themselves that just take it forward and really keep it going […] So, it's sort of driven from within […] I think it works quite well (Site 5)

Older adult participants highlighted the need for activities to be appropriate to their abilities. Ensuring that activity groups worked at the right level for individuals, or having flexibility within session activities so that they felt comfortable to work at their own pace, seemed to lead to positive perceptions of groups:

It's not forced on you, you know, if you can't do a certain thing then just do as much as you can. Which is a good thing rather than having to do something and you feel really out of it and a bit embarrassed (Site 3)

Some instructors and volunteers seemed to be well aware of this need for flexibility, and encouraged people to work within their abilities:

I think the oldest lady in that class is ninety and she doesn't do all my moves, but she does what she can and that's what it's about, you know (P23, Instructor)

In contrast, where it was felt that groups were at too high a level, or where they would be pushed to do more than they felt comfortable with, participants' responses were quite negative:

Well it was embarrassing apart from anything else, as I say, most of the people there seem to be fit, nothing untoward, you know, they can walk miles […] I found it a bit off putting (Site 5)

In this respect, programmes should be mindful of involving older adults of all ability levels in the early stages of programme development to ensure activities also cater for those with lower perceived ability to engage in physical activity.

### Engagement and access of programme activities

A key challenge for deliverers was determining how to attract locally based older adults to activities, particularly inactive older adults. Word‐of‐mouth seemed to have an important role—particularly where information was shared by a trusted source, or by a person to whom older adults related. Being able to relate to, and being similar to, other participants seemed valued by participants: ‘*We're all in the same boat here aren't we?*’ (Site 1). The concept of relatability was turned into a marketing strategy in one area which presented videos utilizing the life experiences of older adults, aiming to present individuals to whom the target audience could relate:

We just wanted a face for the campaign or like someone—'cos, you know, I guarantee they'll probably get so many more people speaking to them when it's a relatable kind of person […] a relatable kind of more trusted person (P22, Co‐ordinator)

Older adults made valuable suggestions about the development and placement of marketing materials. This involvement seemed to create a sense of ownership and responsibility towards the group, with some people taking the initiative to distribute leaflets themselves – a strategy which seemed to increase engagement:

They've taken leaflets to put in, for example, local chippy […] we gained two or three participants from just picking that stuff up, […] local shops, hairdressers (P27, Co‐ordinator)

Many older adults spoke of commitments which could affect their ability to regularly attend sessions (e.g. work, caring, appointments). Individuals therefore appreciated being able to ‘pay as you go’:

I do like the fact that I don't have to commit myself every week because there are other things to be doing as well (Site 1)

Some older adults lacked confidence to attend groups alone, and having someone attend with them for a first session could encourage initial engagement: ‘*I didn't have the courage to come on me own, so she came with me*’ (Site 2). Where projects involved peer mentor volunteers, providing such support could be positively perceived:

So the idea obviously is to make her independent so that she goes on her own […] so slowly she is sort of gaining back some of her confidence and independence (P21, Volunteer)

Areas that adopted a place‐based approach, utilizing existing resources in the community seemed attractive to local residents, with being local to participants minimizing transport need and encouraging engagement: ‘*this is across the road so I couldn't not could I really*’ (Site 1). Such an approach could also allow activities to take place with minimal cost:

Because if there's no funding and that's a building that can be used during the day to facilitate those clubs, then I think that's great (Site 5)

Physical proximity could also help to overcome some psychological barriers:

To have things on offer that are fairly local to them […] although it may feel a bit challenging, doesn't feel like it's too far out of their comfort zone (P21, Volunteer)

An issue that was highlighted is that the age range targeted by the GM‐AA programme was very wide – for those aged 55 years and older. There was a sense that many GM‐AA activities were targeting the older age range (e.g. 65+ years), and it is important to also understand how to meet the needs of younger older adults.

Predominantly the timing of sessions, you know, has probably led towards more the older end of being an older adult. […] not really touched on sort of fifty‐five to sixty‐five, those in work. And I think it's basically to do with, 1) the offer and the perceptions of ageing. And that, you know, fifty‐five isn't old. […] you perhaps wouldn't perceive yourself in that cohort of people (P33, Coordinator)

Including the views of ‘younger’ older adults, and those still in employment in activity co‐design and development, may therefore be beneficial to ensure there are opportunities for people to maintain activity at any age both pre‐ and post‐retirement, and address barriers such as sessions taking place within ‘working’ hours.

### Enjoyment and perceived benefits of sessions

Groups which offered varied activities within sessions enabled participants to select activities they enjoy rather than feeling forced to engage in a specified activity.

The fact that there's so many activities to choose from. Like last week we did yoga and there was one man that didn't want to do it so he went for a walk. So you don't just have to do that activity that's on for that week, there's plenty to choose from (Site 5)

This flexible approach to participation and the laid‐back atmosphere it appeared to create was valued by participants, seemingly emphasizing the enjoyability of sessions: ‘*It's all a healthy bit of fun really*’ (Site 5).

The social element of sessions was highly valued by older adults, and recognized by deliverers as being valuable:

It's very important [the social element of group]. If people didn't talk I probably wouldn't keep on going (Site 5)

The camaraderie of playing team sports seemed to be enjoyed by the walking sports groups (Sites 2 and 4).

Beyond the social aspect, participants also felt they benefited from the support and encouragement of those around them. This appeared to create a sense of community among participants:

I think it's lovely the way the people all mix and socialise and have time for each other […] a lot of support and a lot of friendship and a lot of caring […] [People] do look after each other […] It's that sort of community really, isn't it? (Site 5)

Individuals supported each other outside of sessions, showing concern for each other's wellbeing:

Well, it's like, there's one, one person missing today […] Now I'm going to send her a note […] you know, you can be a bit concerned […] you think, well, I wonder why she's not turned up (Site 2)

Enjoyment of the sessions seemed to enable people to do more activity, more easily than they would have expected, as individuals were not thinking about the exercise aspect:

So it's like almost unconscious exercise, because you don't think you're exercising ‘cos you're just playing (Site 2)

Similarly, enjoyment of music seemed to reduce individuals' focus on the exercise aspect of an activity, as well as encouraging movement of itself: ‘*music it gets everybody doesn't it?*’ (Site 1).

The music plays a great big part because if they recognise the music then they'll go “Oh hasn't that hour gone fast” (P23, Instructor)

### Support needs of individuals delivering activities

Evidence of training and supportive networks around the new ways of working were apparent for some staff working in or trained by the programme. In one MBC, volunteers were trained to deliver physical activity sessions to groups, and the high quality of this training was appreciated. Elsewhere, a volunteer expressed a desire for repeats of a recently held meeting, in order to increase peer contact:

You need that kind of support […] social meetings with other people in the scheme so that (a) you don't feel that you are on your own and (b) if there are any other issues regarding perhaps boundaries (P29, Volunteer)

Empathy and listening skills were recognized as important for activity providers. However, working closely in this way with individuals could lead to deliverers learning of older adults' additional support needs beyond the programme scope. All individuals delivering services, including volunteers, need training and support to appropriately manage such situations while maintaining healthy boundaries. Even experienced providers faced times where older adults sought support outside of deliverers' physical activity role: ‘*Sometimes they do phone you while you're at home*’ *(P23, Instructor)*.

There seems to be a need to ensure that volunteers feel well supported by activity providers, as there is potential for some to feel isolated in their roles which added a feeling of pressure to the delivery:

So I fall down and I break my hip, who's going to do it next [activity day]? […] It's just you (P25, Volunteer)

There was also a sense that volunteers could feel under‐valued and would welcome recognition of the work they were carrying out. How individuals feel about their volunteer role might depend on both the type of role they are carrying out (i.e. whether they are delivering sessions or taking a supporting role), and their own situation in life. One co‐ordinator observed that people's financial and health status might affect their ability to become involved:

Is it because they, you know, maybe have a better pension, long‐term health condition free? Does that allow you to be that older adult champion within a community, against those that might aspire to be or those that are less engaged because it might be that, actually, they are restricted because they have a long‐term health condition? (P33, Co‐ordinator)

Involving older adult volunteers in supporting and delivering physical activity sessions may facilitate the sustainability of programmes, especially when funding is scarce. However, it would seem vital to provide a support system to volunteers to ensure that they can access training and support, and feel valued.

## DISCUSSION

Overall, the views of both older adult participants and service deliverers suggest that the physical activity sessions that were developed using participatory approaches were enjoyable and met the needs of older adults participating in the sessions. It seems, therefore, that using participatory approaches in intervention development may help to ensure acceptability of such programmes. However, it was noted as important that co‐design is inclusive of a diverse range of older adults, to ensure programmes are accessible to all groups, including those still in employment, and those with lower confidence to be active. It also seemed that involving older adults in project development may have contributed to a sense of ownership and responsibility towards the group, which could lead to a level of self‐sufficiency. Making use of people's experiences and relatability in marketing may be a promising approach in recruiting older adults to activities. The role of peer mentors in supporting individuals to access groups was perceived positively, and place‐based approaches seemed to help individuals overcome both physical and psychological barriers to access activities in their local communities. However, in a theme reflecting deliverers' perspectives, volunteers discussed the need for more training and support, and the need to feel appreciated within their role.

Previous research has identified that older adults can be indifferent or even hostile towards the idea of participating in physical activity for its own sake (McGowan et al., 2018), and may instead be motivated by a desire to increase social contact, and to take part in enjoyable activities (Devereux‐Fitzgerald et al., 2016). The present research builds on this, suggesting that the use of participatory approaches in intervention development and implementation may lead to physical activity interventions which are acceptable and in which individuals feel motivated to participate. Importantly, working collaboratively with older adults in the design of activities appears to be valuable in ensuring that physical activity programmes meet their goals of enjoyment and socialization, resulting in good levels of acceptability.

Previous research has also found that older adults desire dedicated programmes and spaces for their age group that are easily accessible within their local communities (McGowan et al., 2018). The development of place‐based approaches in the GM‐AA programme appears to have facilitated this, by drawing on local assets to provide accessible spaces dedicated to provision for older adults. Importantly, this place‐based working has been shown to work well in areas that are typically more deprived, where community provision for older adult groups is often limited (McGowan et al., 2019).

Previous research has identified the importance of peer volunteering and the positive impact of such schemes on physical activity related outcomes in older adults (Kritz et al., 2023; Stathi et al., 2020). Similarly, a systematic review found a positive association between leisure‐time physical activity and social support (Lindsay Smith et al., 2017). These findings are in line with the current study's indication that participants valued support from peer mentors. However, the present study also highlighted the importance of ensuring good support for volunteers themselves, with the potential for older adults in voluntary roles to feel unsupported and under‐appreciated. Indeed, a recent study identified perceived lack of support as a reason for dropout among peer volunteers in a walking intervention for older adults (Kritz et al., 2021). The findings of our previous paper suggested that involving older adults in the design and development of activities could be seen to facilitate sustainability of the programme beyond the funding period, without staff involvement (Davies et al., 2021). The present study, however, shows that involving older adults is unlikely to be sufficient to ensure sustainability in the delivery of the programme of itself, as continued support for volunteers is needed.

These findings are also consistent with relevant theories in this area. In particular, theories such as the Dual Mode Theory of physical activity emphasize the importance of affect in physical activity engagement (Ekkekakis, 2003). This theory proposes that cognitive factors are highly influential in affective responses to physical activities that are typically lower or moderate intensity (which many GM‐AA activities would be categorized as). In the present findings, cognitive factors such as increased confidence appeared to be of importance to engagement and experience of the programme, and participants spoke of their enjoyment of the activities within the programme. This is in line with recent research showing that specific positive psychological and cognitive constructs can both promote and result from physical activity (Millstein et al., 2020).

### Strengths and limitations

This study provides valuable in‐depth insight into how older adults and service deliverers experienced community physical activity interventions designed using participatory approaches with respect to implementing and participating in those interventions. Experiences were explored across multiple intervention sites within a large, diverse conurbation. It is, however, important to acknowledge that this research was conducted in an age‐friendly region (as recognized by the World Health Organization (N.D.)) with a focus on physical activity. Nevertheless, the current study included some of the most deprived localities in England. It may be that less deprived areas would face fewer barriers to implementation of similar programs developed with co‐production techniques. However, it is unknown how implementation of the programme in an age‐friendly and relatively deprived region may have affected findings, therefore replication is warranted in other geographic areas.

Older adult participants who agreed to be interviewed may have been keen attenders of the programme; we may not have captured the perspectives of those who only attended one or two sessions, whose views may have differed. However, including the views of service deliverers in this multi‐perspective analysis may have helped to shed light on issues with the wider group of older adults attending activities, as deliverers may have had insight into views of those who only attended a small number of sessions. We did not measure physical activity levels of the present sample, but such measurements were taken as part of the wider programme evaluation. These data showed that, of 228 participants retained in the programme at six‐month follow‐up, 71% were categorized as inactive at baseline (French, Davies, et al., 2020), using Sport England's Short Active Lives Survey (Milton et al., 2017). Therefore, those who kept attending the programme were not necessarily physically active to begin with.

With respect to ethnicity, the GM‐AA programme was largely representative of the population of adults aged 55 years and above in Greater Manchester, with 87% of those taking part in the programme describing their ethnicity as ‘white’ (French, Davies, et al., 2020). This is a strength of the programme as a whole. However, in the present study sample there was only one ethnic minority service deliverer and no ethnic minority older adult participants who had taken part in the GM‐AA activities; therefore, a limitation of the current study is that ethnic minority perspectives of the GM‐AA programme have not been well captured.

### Implications

The findings presented offer multiple implications for the development and delivery of physical activity promotion programmes for older adult populations. Importantly, the findings demonstrate that using participatory approaches without intensive involvement of researchers (as has been the case in the past) can result in activities which can be acceptable and appealing to older adults. This has important implications in terms of implementing such programmes at scale while minimizing resource and capacity burden.

Older adults were seen as assets in terms of the skills and knowledge they could bring to the projects. For example, utilizing older adults' experiential knowledge was valuable in developing marketing strategies. Further, including older adults in visual marketing (e.g. through video) could foster a sense of relatability to the target audience and challenge the notion that physical activity is incompatible with ageing (McGowan et al., 2018). In future, utilization of such asset‐based strategies may facilitate recruitment of individuals who would not traditionally engage, e.g. inactive older adults who typically feel negative towards the idea of engaging in physical activity (McGowan et al., 2018).

Local provision and utilization of local assets for local needs was typically preferred. Place‐based approaches should focus on determining what assets are already in place in local communities and how to build on these assets. Such an approach may minimize additional costs and produce more sustainable physical activity programmes that cater for local needs.

For volunteers involved in activity delivery, who could themselves be older adults, the need to feel valued and supported was identified. Future programmes should ensure that there is appropriate support for these volunteers (e.g. by allocating time to discuss issues with programme staff) and explicitly recognizing volunteers' contributions to programmes could be valuable. This should reduce the risk of dropout of volunteers in such initiatives (Kritz et al., 2021).

## CONCLUSIONS

The current study offers evidence that place‐based physical activity programmes for older adults developed using participatory approaches can be acceptable both to service deliverers and individuals participating in activities. Having older adults involved in the planning and running of activities appears to be a promising avenue and may facilitate their taking ownership of groups and activities. This may contribute to the sustainability of such programmes, but it is important to ensure that ongoing support is provided, and so longer‐term resources should not be overlooked.

## AUTHOR CONTRIBUTIONS

**Laura J. McGowan:** Project administration; writing – original draft; investigation; formal analysis; writing – review and editing; methodology. **Amy Davies:** Project administration; writing – review and editing; investigation; formal analysis; methodology. **David P. French:** Conceptualization; funding acquisition; project administration; writing – review and editing; formal analysis; methodology; supervision. **Angela Devereux‐Fitzgerald:** Writing – review and editing; investigation; formal analysis; methodology. **Elisabeth Boulton:** Funding acquisition; project administration; writing – review and editing; investigation; methodology. **Chris Todd:** Conceptualization; funding acquisition; project administration; writing – review and editing; methodology; supervision. **Christopher Phillipson:** Funding acquisition; writing – review and editing; methodology; supervision. **Rachael Powell:** Funding acquisition; project administration; writing – review and editing; investigation; formal analysis; supervision.

## FUNDING INFORMATION

This study was carried out as part of the Greater Manchester Active Ageing programme evaluation. Funding for both the programme and evaluation was provided by Sport England to Greater Sport, who subcontracted this evaluation. LM was also funded by the Economic and Social Research Council as part of the North‐West Social Science Doctoral Training Partnership (Grant 7325648).

## CONFLICT OF INTEREST STATEMENT

The authors declare that they have no competing interests.

## CONSENT FOR PUBLICATION

All participants provided written consent regarding participation in this research, including providing explicit consent for quotations to be published.

## Supporting information


File S1.

File S2.

## Data Availability

The anonymised dataset analysed during the current study is available from the corresponding author on reasonable requests, with restrictions to access to ensure participant confidentiality (as per study approvals).
