# Supplementary material for: Understanding the experiences of older adult participants and individuals involved in the delivery of a physical activity programme based on participatory approaches: A qualitative analysis
Source: Br J Health Psychol. 2024 Sep 23;30(1):e12747. doi: 10.1111/bjhp.12747 (PMC11586820; doi:10.1111/bjhp.12747)
Supplement: Supplementary file 1 — File S1. [file BJHP-30-0-s002.docx]

***A brief overview of new approaches and activities developed across the eight localities***

| **Approaches** |
| --- |
| Approaches used included:   - Working in partnership with organisations such as charities, leisure providers and community and voluntary services - Inviting organisations/entrepreneurs to apply for small investment pots to tackle inactivity - Targeting specific populations – e.g. people with long term health conditions, caring responsibilities, or life changes (e.g. bereavement, retirement); targeting neighbourhoods with high levels of deprivation and physical inactivity - Using older adults as ‘champions’ to support increased activity - Using outdoor centres to offer co-designed and participant-led activities - Training older adult volunteers to deliver physical activity within social groups - Using ‘peer mentors’ to support individuals to attend sessions - Campaigns to increase awareness and encourage physical activity uptake - Drawing on e.g. market research |
| **Example activities** |
| Dancing, cycling, yoga, gardening, chair-based exercise groups, walking, kayaking, indoor games (e.g. curling, darts), walking versions of team sports, tai chi, zumba, introducing physical activity into existing social groups. |
| **Delivered by whom?** |
| Individuals/organisations delivering/supporting activities included: fitness instructors, care homes, local activity clubs/organisations, volunteers (including older adult volunteers), self-facilitation by activity participants. |
| **Locations** |
| Locations included: care homes, community centres, local parks, community buildings e.g. pub; community gardens; leisure centres/sports facilities. |
