# Supplementary material for: Understanding the experiences of older adult participants and individuals involved in the delivery of a physical activity programme based on participatory approaches: A qualitative analysis
Source: Br J Health Psychol. 2024 Sep 23;30(1):e12747. doi: 10.1111/bjhp.12747 (PMC11586820; doi:10.1111/bjhp.12747)
Supplement: Supplementary file 2 — File S2. [file BJHP-30-0-s001.docx]

| Supplementary file 2: Example questions from topic guides | |
| --- | --- |
| ***Participant Group*** | ***Example Questions*** |
| ***Older adult participants***  *(Sites 1-4 focus groups and Site 5 interviews)* | - What attracted you to the GM Active Ageing programme? - What do you think about the programme? - How could the programme be improved? - In what ways do you think the programme has helped you to increase your levels of physical activity (if at all)? - What are the important benefits you get from coming to this group? (e.g. social, physical health, mental health) - Is there anything that worries you about coming to this group? - What do you think might help other people to come to this group or groups like this? |
| ***Service deliverers***  *(Interviews)* | - What is your role in delivering this activity? - What is it about these activities that you think appeals to older adults? What are people’s preferences? - What about physical activities that aren’t working out? Why do you think this is? - How do you think location might affect the success of this GM Active Ageing project? - What would you say is key to successful provision of physical activity for older adults in Greater Manchester? |
